# Supplementary material for: Differential prevalence of pathobionts and host gene polymorphisms in chronic inflammatory intestinal diseases: Crohn’s disease and intestinal tuberculosis
Source: PLoS One. 2021 Aug 18;16(8):e0256098. doi: 10.1371/journal.pone.0256098 (PMC8372915; doi:10.1371/journal.pone.0256098)
Supplement: S1 Fig — (A) Amplicons corresponding to AIEC (Lanes 1–9). Lane M loaded with 100 bp DNA ladder. (B) Amplicons corresponding to L.monocytogenes (Lanes 2–6), Bands in lane 1 and lane M correspond to amplicon of positive bacterial culture and 200 bp ladder resp. (C) Amplicons corresponding to C.jejuni (Lanes 2–6), Bands in lane 1 and lane M correspond to amplicon of positive bacterial culture and 100 bp ladder resp. (D) Amplicons corresponding to Y.enterocolitica (Lanes 1 and 2), Bands in lane 5 and lane M correspond to amplicon of positive bacterial culture and 200 bp ladder resp. (PDF) [file pone.0256098.s001.pdf]

A

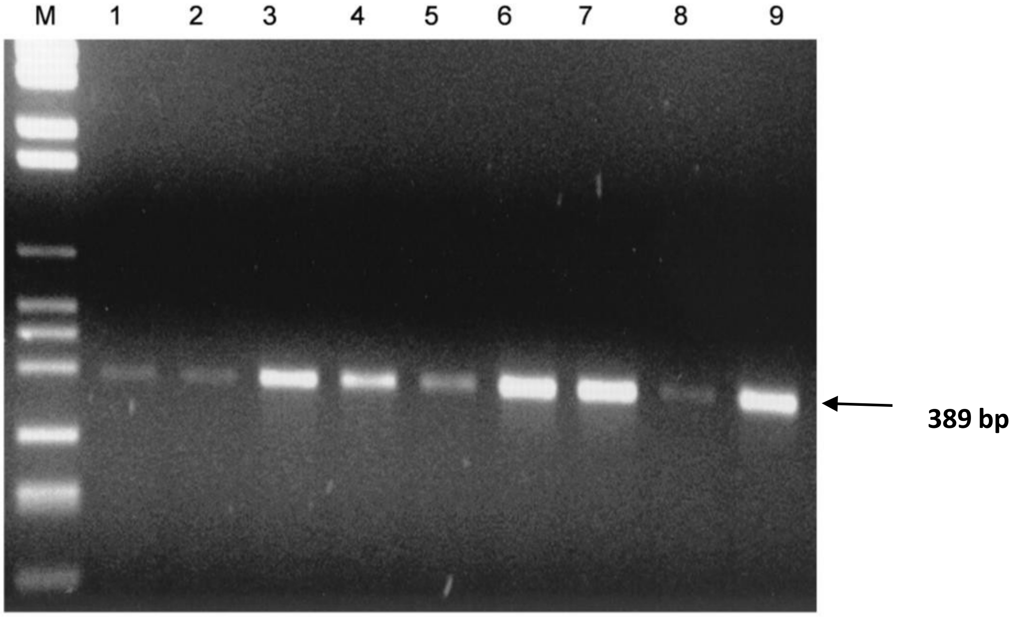

B

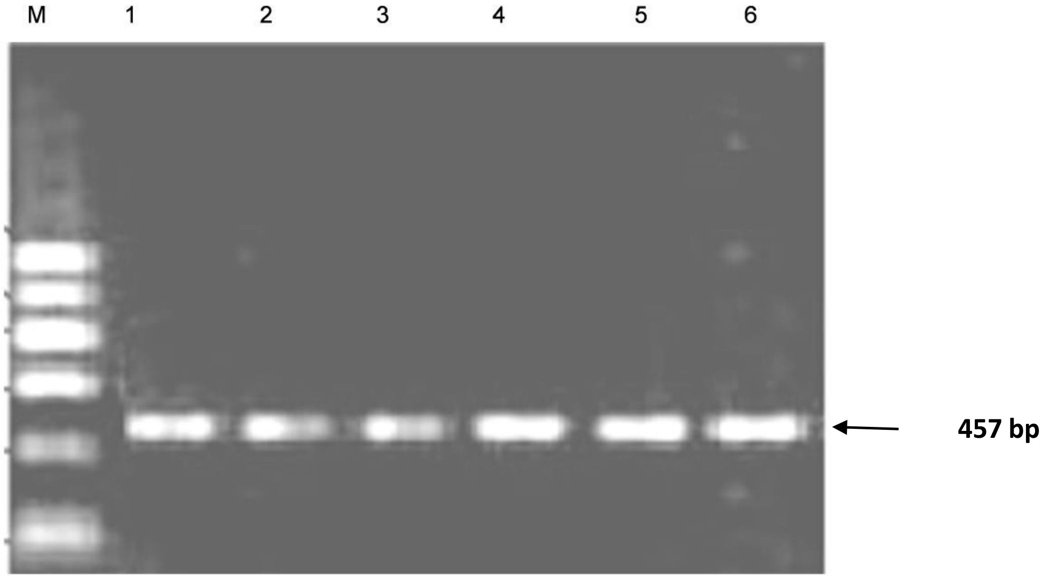

C

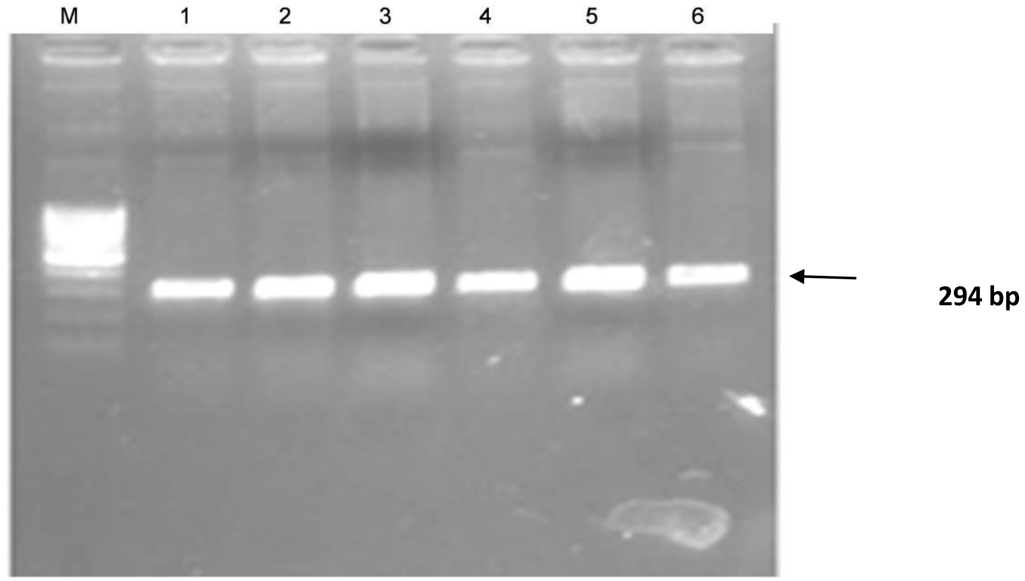

D

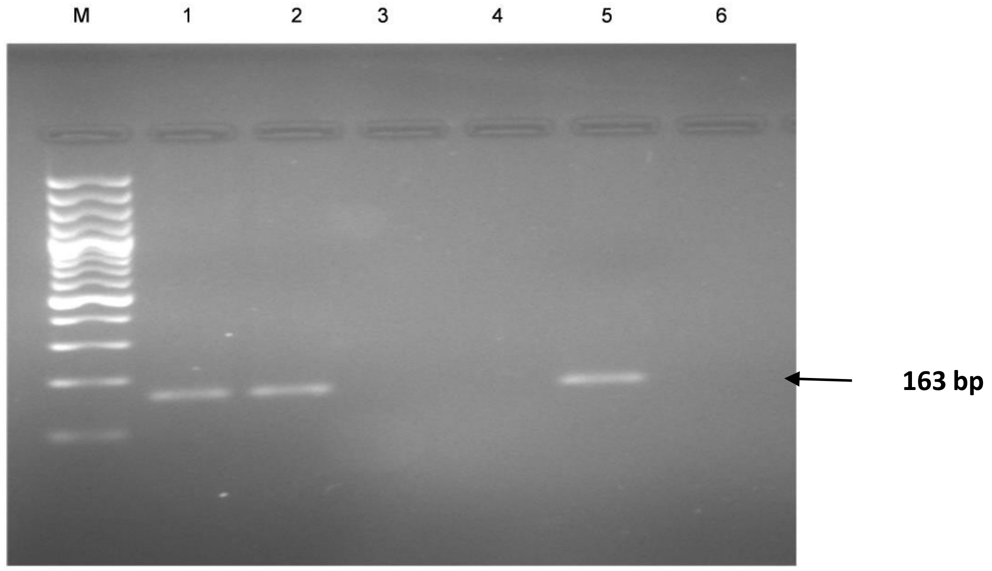

S1 Fig. The raw uncropped agarose gel, obtained post electrophoresis of the qPCR products

(A) Amplicons corresponding to AIEC (Lanes 1-9). Lane M loaded with 100 bp DNA ladder. (B) Amplicons corresponding to *L.monocytogenes* (Lanes 2-6), Bands in lane 1 and lane M correspond to amplicon of positive bacterial culture and 200 bp ladder resp. (C) Amplicons corresponding to *C.jejuni* (Lanes 2-6), Bands in lane 1 and lane M correspond to amplicon of positive bacterial culture and 100 bp

ladder resp. (D) Amplicons corresponding to *Y.enterocolitica* (Lanes 1 and 2), Bands in lane 5 and lane M correspond to amplicon of positive bacterial culture and 200 bp ladder resp
