# Supplementary material for: Differential prevalence of pathobionts and host gene polymorphisms in chronic inflammatory intestinal diseases: Crohn’s disease and intestinal tuberculosis
Source: PLoS One. 2021 Aug 18;16(8):e0256098. doi: 10.1371/journal.pone.0256098 (PMC8372915; doi:10.1371/journal.pone.0256098)
Supplement: S1 Table — a F and R indicate forward and reverse primers, respectively. (DOC) [file pone.0256098.s002.doc]

**S1 Table.** Primers used for detection of selected bacteria in the present study

| **Target bacteria** | **Primer sequence (5’-3’)^a^** | **Target gene** | **Annealing temp (°C)** | **Amplicon size (bp)** | **Refrences** |
| --- | --- | --- | --- | --- | --- |
| Adherent-invasive  *E. Coli* (AIEC) | F, GATCTTTCGACGCAAATC R, CGAGCAGAAACATCGCAG | FimH | 63.4 | 389 | [27] |
| *Listeria monocytogens* | F, ATGTCATGGAATAA  R, AAAAACACCTTGGAAAAGC | iap | 42.9 | 457 | [28] |
| *Campylobacter jejuni* | F, CTGATAAGGGTGAGGTCACAAGT  R, CTTGCTTGTGACTCTTAACAATG | 16S-23S ITS | 61.2 | 294 | [29] |
| *Yersinia enterocolitica* | F, ATGATAACTGGGGAGTAATAGGTTCG  R, CCCAGTAATCCATAAAGGCTAACATAT | ail | 63.8 | 163 | [30] |

^a^ F and R indicate forward and reverse primers, respectively.
