# Supplementary material for: Differential prevalence of pathobionts and host gene polymorphisms in chronic inflammatory intestinal diseases: Crohn’s disease and intestinal tuberculosis
Source: PLoS One. 2021 Aug 18;16(8):e0256098. doi: 10.1371/journal.pone.0256098 (PMC8372915; doi:10.1371/journal.pone.0256098)
Supplement: S2 Table — a F and R indicate forward and reverse primers respectively. b The polymorphic base is in bold; underlined letters indicate mismatched bases. (DOCX) [file pone.0256098.s003.docx]

**S2 Table.** **Allele-specific primers used for genotyping**

| **Gene** | **Primer name^a^** | **Primer sequence (5’ to 3’)^b^** | **Amplicon size (bp)** |
| --- | --- | --- | --- |
| IRGM  rs13361189 (C/T) | Wildtype F | CAAGCAGAGTGTGCTTGAAAAC**C** | 369 |
|  | Mutant F | CAAGCAGAGTGTGCTTGAAAAC**T** |  |
|  | Common R | AGATGCACAAGGGAGTGGAG |  |
| IRGM  rs10065172 (C/T) | Wildtype F | ACCACAACCCTGGAGAACTAA**C** | 167 |
|  | Mutant F | ACCACAACCCTGGAGAACTAA**T** |  |
|  | Common R | CTGAGGTCCATGTCTAGCTT |  |
| IRGM  rs4958847 (A/G) | Wildtype F | ATAGATTTCATTGCCCAATAC**A** | 424 |
|  | Mutant F | ATAGATTTCATTGCCCAATAC**G** |  |
|  | Common R | GTTGCATCAGTGCCAAGAAA |  |
| ATG16L1  rs2241880 (C/T) | Wildtype F | TTACCAGAACCAGGATGAC**C** | 110 |
|  | Mutant F | TTACCAGAACCAGGATGAC**T** |  |
|  | Common R | ACCTCTGCAATCCTGTCTAA |  |
| TNFRSF1A  rs4149570 (G/T) | Wildtype F | ATTGGA AAACAGATCC AGACAC**G** | 378 |
|  | Mutant F | ATTGGA AAACAGATCC AGACAC**T** |  |
|  | Common R | GGGAAGAGTGAGGCAGTGTT |  |

^a^ F and R indicate forward and reverse primers respectively. ^b^ The polymorphic base is in bold; underlined letters indicate mismatched bases.
