# Supplementary material for: Differential prevalence of pathobionts and host gene polymorphisms in chronic inflammatory intestinal diseases: Crohn’s disease and intestinal tuberculosis
Source: PLoS One. 2021 Aug 18;16(8):e0256098. doi: 10.1371/journal.pone.0256098 (PMC8372915; doi:10.1371/journal.pone.0256098)
Supplement: Attachment — Submitted filename: Review for Plos One Article by Khan et.al._PONE-D-21-12204.pdf [file pone.0256098.s004.pdf]

Review for Plos One Article by Khan et.al._PONE-D-21-12204

Overlapping clinical characteristics in Crohn's Disease (CD) and intestinal tuberculosis (ITB) make the differential diagnosis a daunting task for clinicians, especially in case of patients from TB endemic countries like India. Differentiation between these two deadly diseases cannot be done with a standalone evaluation and requires a comprehensive approach which includes the sum of clinical, endoscopic, radiological, microbiological laboratory and culture studies for accurate diagnosis. Most of these approaches bear one or other limitations of low sensitivity and specificity. This dilemma results in misdiagnosis and delay in treatment contributing to increased morbidity and mortality. In this direction, study of both genetic and environmental risk factors may prove to be instrumental in deciphering newer biological markers for efficient diagnosis of CD and ITB. Dysbiosis in human gut microbiome and genetic susceptibility has been now associated with various diseases including CD. Thus, differential prevalence of pathobionts and host gene polymorphisms can serve as good biomarker to differentiate between CD and ITB.

The research article entitled "Differential prevalence of pathobionts and host gene polymorphisms in chronic inflammatory intestinal diseases: Crohn's Disease and Intestinal Tuberculosis" (PONE-D-21-12204) by Khan *et.al* highlights the prevalence of pathobionts like adherent-invasive *Escherichia coli* (AIEC), *Listeria monocytogenes*, *Campylobacter jejuni* and *Yersinia enterocolitica* in Crohn's Disease (CD) and Intestinal Tuberculosis (ITB). Consistent with previous studies, this study reveal a significantly enhanced prevalence of the risk alleles of IRGM (rs13361189, rs4958847 and rs10065172) and ATG16L1 (rs2241880) TNFRSF1A polymorphism (rs4149570) genes with CD.

The manuscript is well written and suitable statistical tools and analysis have been applied to determine the significance of the data. Although, the study is limited with lack of statistically significant data, possibly due to the small sample size as also admitted by authors in the manuscript, the findings from this study are very important especially the prevalence of specific SNPs in genes implicated in IBD, in a North Indian cohort. This study takes the field one step closer to a non-invasive and affordable diagnostic procedure for IBD and may prove to be useful for the follow up studies with a bigger sample size in Indian population. Such investigative studies should be pursued with further experiments involving the colonization of wild type, germ-free, and genetically modified mice with an individual bacterial species or with a combination of bacteria, in order to identify the exact causal bacterial strain or core microbiome and clarify the fate of the gut microbiota in IBD.

The manuscript may be considered for publication after addressing the following comments:

Comments:

1. In the introduction, authors mentioned *Mycobacterium avium* subspecies *paratuberculosis* (MAP) as one of the pathogen associated with CD. Also there is moderately high seroprevalence (23.4%) of MAP in human population of north India (SV Singh *et.al.*, J Biol Sci 2014) so, adding MAP prevalence data in this study would have been useful and enhanced our understanding towards the association of MAP with CD in Indian population. Any specific reasons that authors didn't included it in the study?

2. Refer lines 120-122 "Diagnosis of CD and ITB ………. based on standard clinical, radiological, endoscopic and histological criteria" Authors have provided data only from Behaviour of disease (Montreal Classification), location of disease and site of biopsy (refer Table 1). The other clinical, radiological histological, microbiological data is missing. Considering the overlapping clinical characteristics and knowing that the exclusive features between CD and ITB are caseation necrosis on biopsy, positive smear for acid-fast bacillus (AFB) and/or AFB culture, and necrotic lymph node on cross-sectional imaging in ITB (Kedia et.al.,2019), including supportive data from histology of biopsy samples (if possible) and clinical symptoms (representative

images of endoscopy of selected patients among all the groups) will be helpful in validation of the accurate diagnosis of the patients in this study. Alternatively, multivariable double logistic regression analysis of endoscopic and clinical features should have been performed. (Pls refer to research article by Li *et.al*., 2011, DOI 10.1007/s10620-010-1231-4).

3. Since sample storage conditions can affect the quantification of the target microbial markers especially fast growing *E.coli*, the storage conditions of the clinical samples (Biopsies) before the isolation of the nucleic acid should be described in the methods.

4. Refer lines 183-184 (*C. jejuni* prevalence) & 186-188 (AIEC prevalence). Since the Prevalence data for these two strains is not statistically significant, affirmative statements should be avoided. Sentences can be rewritten for better clarity and avoiding any misunderstanding.

5. Refer to lines 258-260, Pls provide suitable references of the previously published studies from the literature, if any, where the comparative assessment of the prevalence of various pathobionts, between CD and ITB was done. A comparison between their methods & findings with that in the present study will be useful.

6. Refer lines 269-270, The statement "The control and ITB groups were found to have low infection incidences" holds true only for *L. monocytogenes and Y. enterocolitica* as only these were significantly less prevalent in the control and ITB groups as compared to CD patients, whereas there was no significant difference in the incidence of the AIEC and *C. jejuni* between the groups. So sentence need to be modified accordingly.

7. Recent case study of an Asian female patient (Korean) with Crohn's Disease reported her case to be initially misdiagnosed as Intestinal Tuberculosis due to active pulmonary tuberculosis (Park et.al., Korean J Gastroenterol, 2021). Given that India is TB endemic country, it may be worthy to include the active pulmonary tuberculosis in the clinical history of the patient to avoid such misdiagnosis specifically in studies involving comparison of CD Vs. ITB. Also, since the baseline features of gut microbiota after or during anti-TB treatment among ITB patients may differ, it may be worth to mention the ATT treatment in recent past of subjects included in this study. The inclusion and exclusion criteria (especially history of tuberculosis, previous/existing anti-tubercular drug therapy at the time of specimen collection) for various groups (CD, ITB and Controls) under this study have not been defined and should be included in the manuscript.

8. As this study doesn't demonstrate any association between the CD associated genetic polymorphisms and the prevalence of various pathobionts, its implications in pathogenesis of CD and ITB should have been discussed in correlation with clinical symptoms of patients under different groups in this study. This would have provided more insights to understand the role of these marker genes in etiology of CD and ITB.
